# Fatal interstitial lung disease associated with Crizotinib pathologically confirmed by percutaneous lung biopsy in a patient with ROS1-rearranged advanced non-small-cell lung cancer: a case report

**DOI:** 10.1186/s12890-018-0682-9

**Published:** 2018-07-20

**Authors:** Shibo Wu, Kaitai Liu, Feng Ren, Dawei Zheng, Deng Pan

**Affiliations:** 1 Department of Respiratory Medicine, Lihuili Hospital, Ningbo Medical Center, No. 57, Xin’ning Road, Ningbo, 315041 China; 2 Department of Radiation Oncology, Lihuili Hospital, Ningbo Medical Center, Ningbo, 315041 China; 3 Department of Radiology, Lihuili Hospital, Ningbo Medical Center, Ningbo, 315041 China; 4 Department of Thoracic Surgery, Lihuili Hospital, Ningbo Medical Center, Ningbo, 315041 China; 5 Department of Diagnosis, Ningbo Diagnostic Pathology Center, No. 79, Huan’cheng Road, Ningbo, 315021 China

**Keywords:** Interstitial lung disease, Crizotinib, ROS1 rearrangement, EGFR mutation

## Abstract

**Background:**

Crizotinib is a multi-target inhibitor approved for the treatment of advanced non-small-cell lung cancer patients with a ROS1 rearrangement. However, interstitial lung disease is a rare but severe and fatal side effect of crizotinib that should lead to immediate discontinuation of the drug. Unfortunately, the pathophysiology, molecular mechanism and risk factors for crizotinib-induced interstitial lung disease remain poorly understood.

**Case presentation:**

We first identified and reported interstitial lung disease induced de novo by crizotinib in a 47-year-old female patient who was diagnosed with advanced lung adenocarcinoma with a ROS1 rearrangement in a malignant pleural effusion. Subsequent next-generation sequencing analysis revealed both ROS1 rearrangement and an EGFR exon 19 deletion mutation in lung biopsy specimens, which were histologically confirmed to be interstitial lung disease. Although crizotinib treatment was ceased immediately and a shock treatment with high-dose methylprednisolone as well as other necessary treatment procedures was applied to reverse the interstitial lung disease process, the patient died.

**Conclusions:**

The present case indicates that while treating non-small-cell lung cancer patients with crizotinib, it is important to constantly monitor any newly emerging respiratory symptoms and unexplained imaging changes, which may suggest an adverse effect related to drug-induced interstitial lung disease or even lethality. Histopathology and molecular pathological examination of lung biopsy specimens may help clinicians understand the development mechanism and exclude other causes.

## Background

Crizotinib is a multi-target inhibitor, which was granted a full approval by the Food and Drug Administration (FDA) for the treatment of advanced non-small-cell lung cancer (NSCLC) patients with a ROS1 rearrangement in March 2016. However, interstitial lung disease (ILD) is a rare but severe and fatal side effect of crizotinib that should lead to immediate discontinuation of the drug. Unfortunately, the pathophysiology, molecular mechanism and risk factors for crizotinib-induced ILD remain poorly understood. Here, we describe a case of SDC4-ROS1 rearrangement-positive advanced lung adenocarcinoma with de novo crizotinib-induced ILD.

## Case presentation

A 47-year-old female Chinese patient was admitted to our hospital in January 2018 due to complaints of continuous cough and a feeling of breathlessness for more than a week. The patient did not have a history of alcohol consumption or smoking. She refused to reveal a special occupational history and the medical history of her family.

**Fig. 1:**
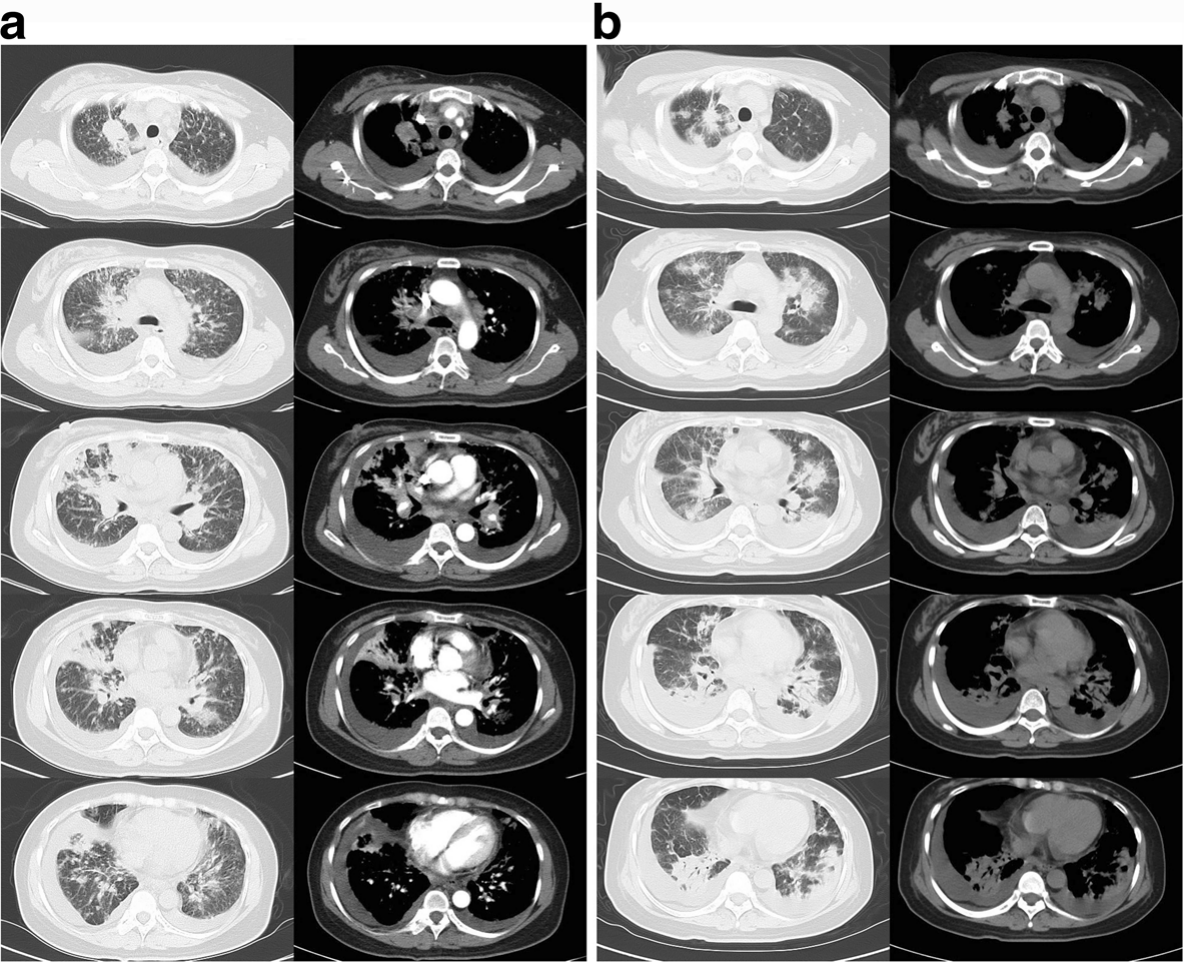
**a**. Prior to treatment with crizotinib, a chest CT scan revealed a large irregularly shaped mass on the upper right lobe, accompanied by multiple nodules, plaques and consolidated masses of diverse sizes randomly distributed in both lung fields. Nodular thickening of the interlobular septa and fissures, which suggested lymphangitis carcinomatosa, hilar and mediastinal lymphadenopathy, and bilateral pleural effusions, were identified as well. **b** Ten days after the initiation of crizotinib, chest CT revealed a significant shrinkage of intrapulmonary neoplastic lesions, lymphadenitis and lymphadenectasis but not of multiple new-onset ground-glass opacities and consolidation lesions throughout both lungs

A chest computed tomography (CT) scan revealed a large, irregularly shaped mass on the upper right lobe, accompanied by multiple nodules, plaques and consolidated masses of different sizes, randomly distributed in both lung fields. Nodular thickening of the interlobular septa and fissures, which suggested lymphangitis carcinomatosa, hilar and mediastinal lymphadenopathy and bilateral pleural effusions, was identified by the CT scan as well (Fig. 1a).

An immediate drainage was conducted for the right pleural effusion, followed by a series of tests. Methylprednisolone (MP) at 80 mg/day was administered to alleviate dyspnoea associated with lymphangitis carcinomatosa. With oxygen therapy via a nasal catheter at a flow rate of 6 L/min, her arterial blood gas was measured to have values of a PaO_2_ of 55.0 mmHg, a PaCO_2_ of 32.0 mmHg, and a pH of 7.49. The carcinoembryonic antigen (CEA) level in hydrothorax was 7.5 μg/L (normal 0–5 μg/L), whereas the serum CEA level was 12.4 μg/L. The rest of the important blood and sputum test indicators are described in Table 1.

**Table 1:** The rest important blood and sputum testing indicators of pre- and post-treatment with Crizotinib

	Blood testing indicators							Sputum indicators
	Carbohydrate antigen 199 (IU/ml)	Cytokeratin fragment (ug/L)	Neuron-specific enolase (ug/L)	Squamous cell carcinoma antigen (ug/L)	White blood cell count (× 10^9^/L)	Erythrocyte sedimentation rate (mm/h)	C-reactive protein (mg/L)	Sputum culture
Normal range	0.0~ 37.0	0.00~ 7.00	0.00~ 10.00	0.00~ 2.50	3.5~ 9.5	0~ 20	0~ 8	NA
Pre-treatment	100.8	14.31	4.85	22.94	11.3	32	95.1	negative
Post-treatment	57.3	15.14	5.84	7.89	16.7	10	4.1	negative

**Fig. 2:**
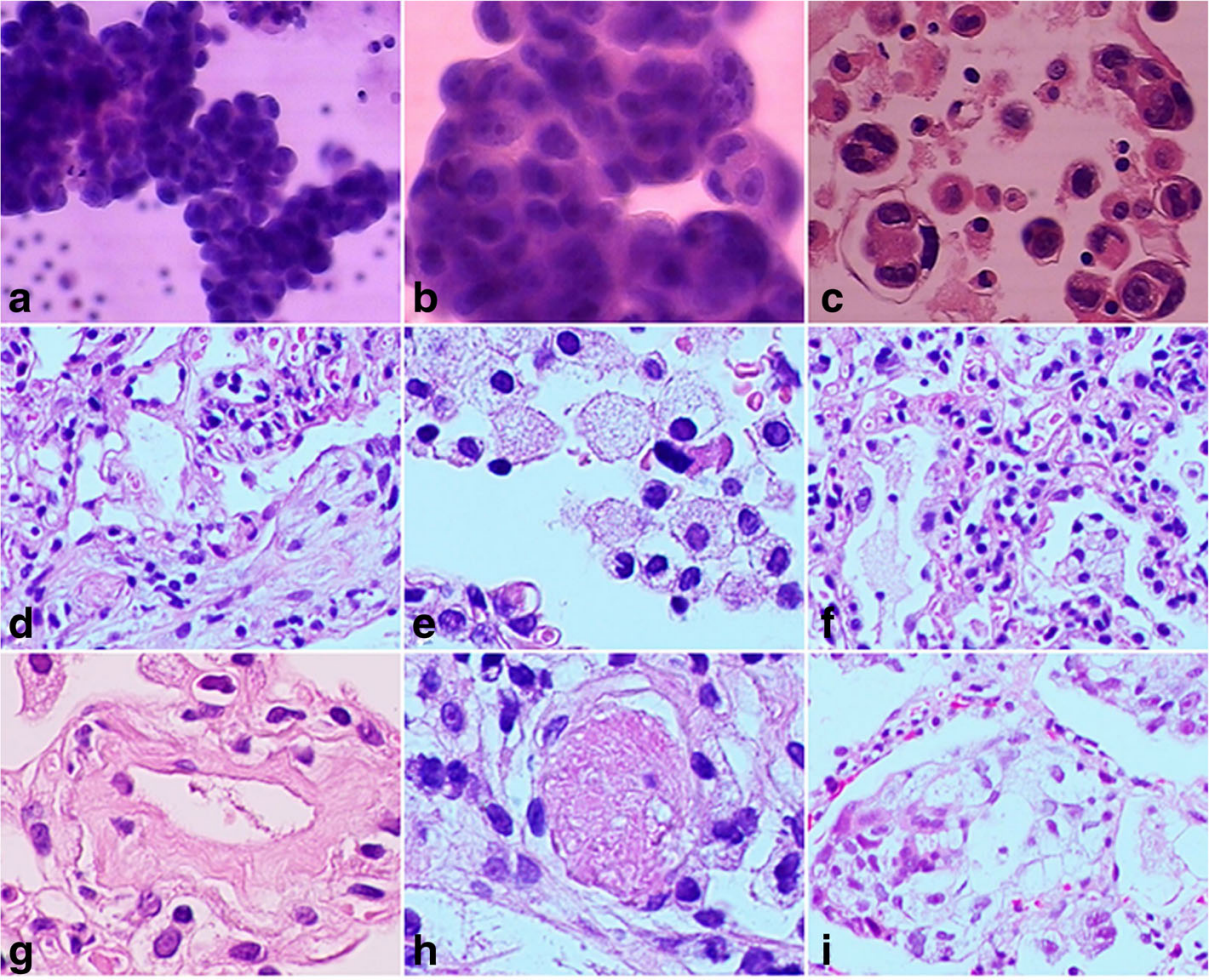
**a**-**c** HE staining (× 200), (× 400), (× 200), liquid-based cell smear and a cell wax block revealed that tumour cells with gigantic nucleoli were distributed in clusters and presented with a round-edge, strongly encapsulated, obviously three-dimensional structure. Fig. 2**d**-**l** HE staining showed histological characteristics of acute interstitial pneumonia. Fig. 2**d** (× 200) Diffuse alveolar oedema, thickening of the septa. Fig. 2**e** (× 400) Macrophages or foamy macrophages prominently present in differently sized alveolar spaces. Fig. 2**f** (× 200) Mild infiltration of inflammatory cells in the interstitium. Fig. 2**g** (× 400) Rare and atypical hyaline membrane formations in alveolar spaces. Fig. 2**h** (× 400) Multiple hyaline thrombi (microthrombi) in pulmonary arterioles of partial areas. Fig. 2**i** (× 400) Atypical hyperplasia of type II alveolar epithelial cells in localized areas

With a poor performance status (PS = 4), the patient was unable to withstand tissue biopsy acquisition. A great number of tumour cells positive for thyroid transcription factor-1 (TTF-1) and cytokeratin 7 (CK 7) were confirmed by pathological haematoxylin-eosin (HE) staining examination of hydrothorax, combined with immunohistochemical staining. These observations led to a diagnosis of advanced lung adenocarcinoma with extensive dissemination in the chest (Fig. 2a-c).

**Fig. 3:**
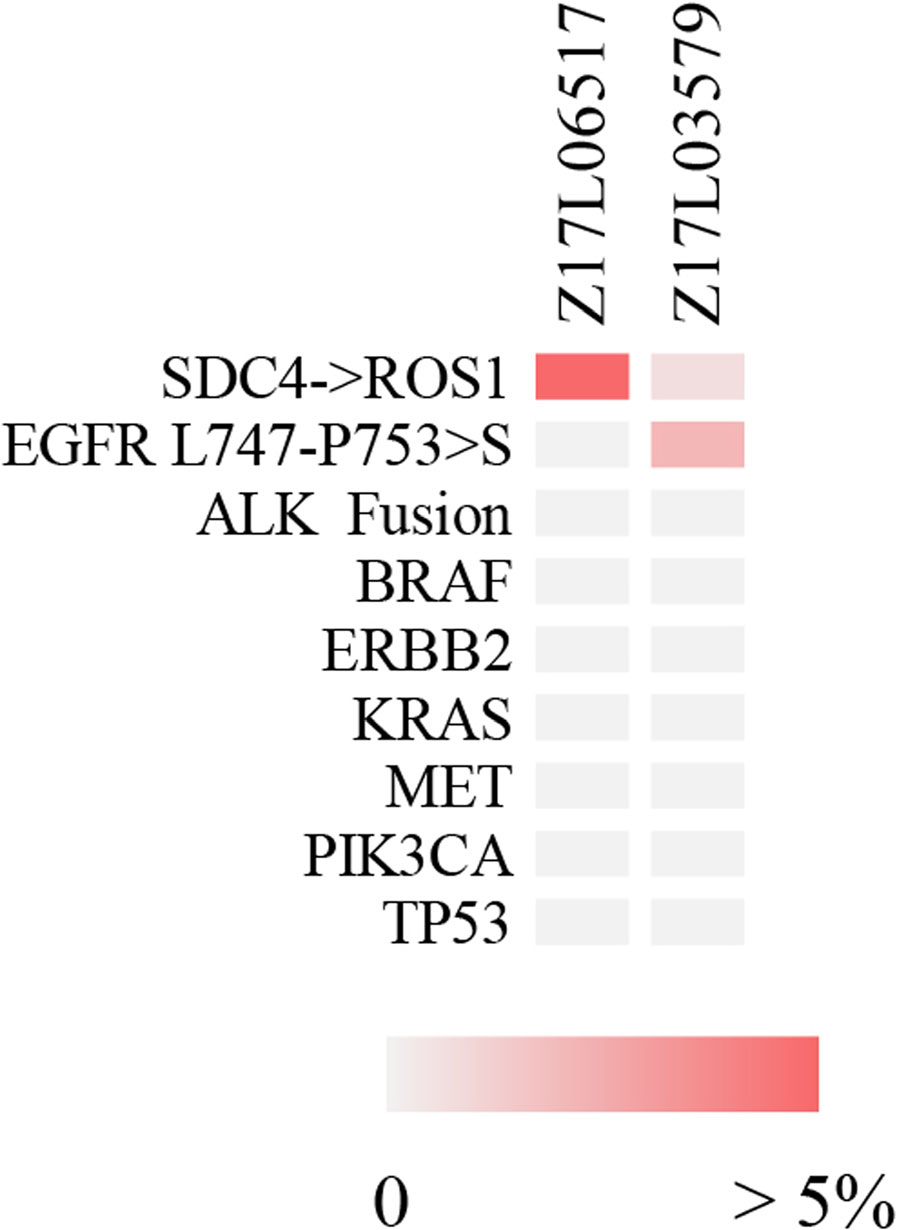
Somatic mutation matrix of genes screened using a circulating single-molecule amplification and resequencing technology (cSMART) panel. Each solid bar in the matrix represents a somatic mutation of a gene (row) in the tested sample (column), with colour codes including light grey for no somatic mutation and red for the presence of a somatic mutation, as indicated. Alternative allele fractions of somatic mutations are labelled with a different colour depth, as indicated by the colour key at the bottom of the figure. Z17L06517: pretreatment NGS. Z17L03579: post-treatment NGS

**Fig. 4:**
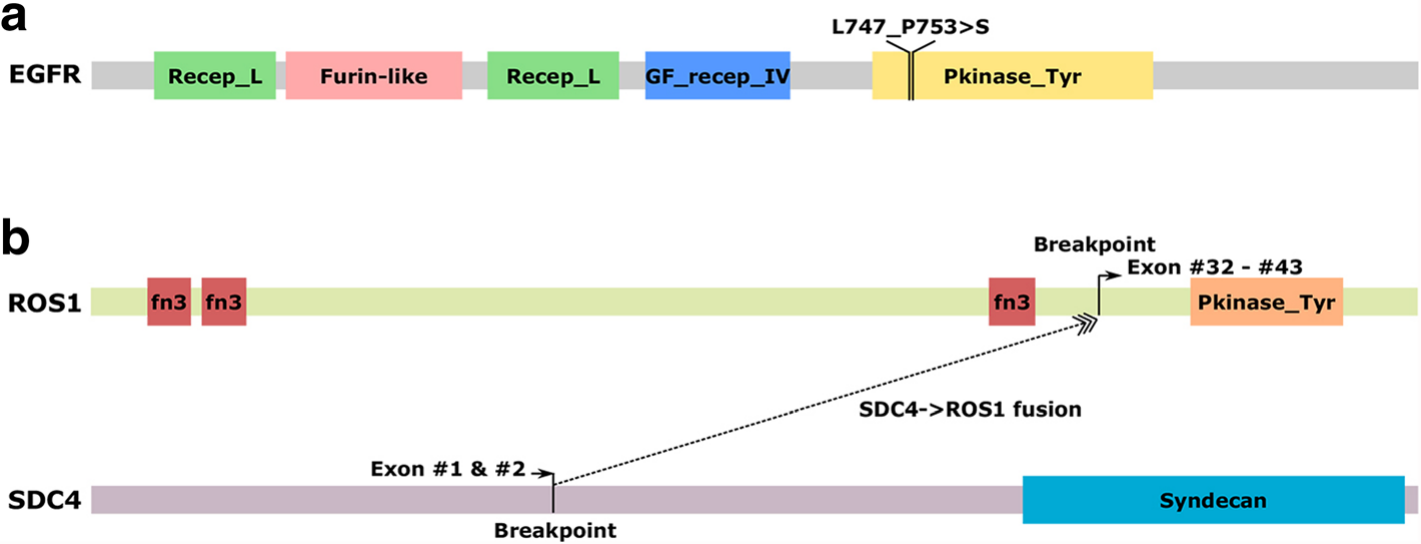
Genomic organization of somatic alterations identified in our patient. **a** complex indel lying in EGFR exon 19, which affects the protein tyrosine kinase domain. **b** Translocation resulted in a fusion product with exons 1 and 2 of SDC4 and exons 32–43 of ROS1, including the protein tyrosine kinase domain. Abbreviations of protein domains: Recep L, receptor L domain; Furin-like, furin-like cysteine-rich region; GF recep IV, growth factor receptor domain IV; Pkinase Tyr, protein tyrosine kinase domain; fn3, fibronectin type III domain; Syndecan, syndecan domain

Next-generation sequencing was then conducted on tumour cells of hydrothorax. The SDC4-ROS1 fusion gene was detected at an abundance of 19.8% in the malignant pleural effusion (MPE). Other mutations, such as those in the EGFR, ALK, KRAS, BFAF, HER2, PIK3CA, MET, and RET genes, were not detected (Fig. 3 Z17L06517, Fig. 4b). The patient was thus orally administered crizotinib at a dose of 250 mg twice per day. After three days of crizotinib treatment, the orthopnoea was greatly relieved, and MP medication was withdrawn. However, oxygen therapy was still required within a one-week time frame of administration. On the tenth day of medication, the patient had a low-grade fever and slight aggravation of dyspnoea. A chest CT re-scan revealed a significant shrinkage of intrapulmonary neoplastic lesions, lymphadenitis and lymphadenectasis. However, multiple new-onset ground-glass opacities and consolidations were detected throughout both lungs (Fig. 1b). The patient was then additionally treated with cefoperazone/sulbactam to exclude the possibility of infection.

In addition, a re-examination of blood tumour markers, infection-related indicators, and characteristics of hydrothorax, combined with percutaneous lung biopsy, was undertaken to clarify the cause. CEA in the pleural fluid and serum increased to 13.6 μg/L and 52.7 μg/L, respectively, after crizotinib medication. Other important indicators are described in Table 1. Consistent with the CT scan, the pathology of hydrothorax suggested a significantly reduced number of tumour cells. Three columnar specimens of percutaneous biopsy from the new-onset consolidation area on the lower left pulmonary were collected promptly, all of which were 1 cm in length and 18 gauge in diameter. Histological observation of these biopsies revealed a diffuse alveolar oedema, thickening of the septa (Fig. 2d), macrophages or foamy macrophages prominently present in differently sized alveolar spaces (Fig. 2e), mild infiltration of inflammatory lymphocytes, monocytes, fibroblasts, and myofibroblasts in the interstitium (Fig. 2f), rare and atypical hyaline membrane formations in alveolar spaces (Fig. 2g), multiple hyaline thrombi (microthrombi) in pulmonary arterioles of partial areas (Fig. 2h), and atypical hyperplasia of type II alveolar epithelial cells in localized areas (Fig. 2i). There was no evidence of infections and invasion of tumour cells. All these pathological changes were consistent with acute lung injury. The pathological stage was assumed to be a transition from exudation to organization. The patient denied having previous pulmonary-related diseases, and there were no other drugs that might potentially cause lung toxicity during crizotinib treatment. Therefore, we made a diagnosis of crizotinib-induced ILD.

MP pulse therapy (0.5 g once per day) was immediately substituted for the original crizotinib treatment and applied for three days. Tracheal intubation and mechanical ventilation were also undertaken because of progressive deterioration leading to respiratory failure. All necessary and additional treatment procedures were conducted to prevent ILD, but the patient died 20 days after the first administration of crizotinib. Upon approval of her family, next-generation sequencing analysis of a lung biopsy sample was performed, which revealed a de novo exon 19 deletion mutation in EGFR, not detected in MPE before treatment with crizotinib (Fig. 3 Z17L03579, Fig. 4a). On the other hand, the frequency of ROS1 rearrangement decreased after crizotinib treatment (Fig. 3). The occurrence of this phenomenon deserves special consideration and investigation of current crizotinib treatment in NSCLC patients.

### Ethics, consent and permissions

Written informed consent has been obtained from the patient’s family for the publication of this case report and accompanying images.

## Discussion

In this study, we report the first case of fatal crizotinib-induced ILD in a ROS1-positive NSCLC patient. ROS1 rearrangements are identified in 1 to 2% of patients with NSCLC [1]. Up to now, crizotinib is still the only targeted agent approved for NSCLC patients with ROS1 rearrangements. A retrospective review of 4 PROFILE clinical trials indicated that the overall incidence of crizotinib-induced ILD was 1.2%, but its mortality rate was up to 50% [2]. It is a rare but serious adverse event in patients on crizotinib therapy.

Although crizotinib-induced ILD in ROS1-positive NSCLC patients has not been systematically characterized before, patients with ROS1 rearrangements had a similar incidence (1.9%, 1 of 53) of ILD as those containing ALK rearrangements (1.7%, 2 of 119) in a phase I PROFILE 1001 study of crizotinib [3]. In addition, the safety profiles of crizotinib are similar for ALK-positive and ROS1-positive NSCLC patients [4]. The factors contributing to crizotinib-induced ILD in ROS1-positive patients remain unclear, whereas risk factors significantly correlated with the development of ILD in ALK-positive NSCLC patients have been described and include age, poor performance status, smoking status, past/concomitant ILD and concomitant pleural effusion [5]. In this case report, a compromised performance status, lymphangitis carcinomatosa and bilateral pleural effusions were observed in the chest and, hence, could be the key factors contributing to the development of ILD.

The median duration from the initiation of crizotinib therapy to the onset of ILD was found to be 23 days (range: 3–763 days) [2]. Créquit et al. described that a severe, usually fatal ILD developed within the 1st month of treatment, and its chest CT manifestation included an early onset of ground-glass opacity, which diffused and spread rapidly in both lungs [6]. In our case, in addition to multiple ground-glass opacity lesions, multiple consolidation lesions were found simultaneously, which coincided with our pathological staging features of the transition from exudation to the organizing period. Moreover, it was reasonable to assume that the clinical symptoms and imaging changes of interstitial pneumonia would be observed earlier than the tenth day without MP administration at the time of admission.

Possible pathological causation of crizotinib-induced ILD has been discussed in several studies [6–8]. In these studies, specimens were obtained either from bronchoscopic lung biopsy, alveolar lavage fluid or corpse biopsy. Limited by the quality and phase of biopsy specimen collection, these pathological findings could only reveal incomplete characteristics of interstitial pneumonia. To explore the pathology of the case reported in this study, we applied an HE staining technique to three freshly coloured, cylindrical percutaneous biopsy samples, which fully met the requirements for diagnosis of ILD. We first excluded the possibility of infection, and no invasion of tumour cells could be observed either. Histological characteristics, such as a thickened alveolar septa, infiltration of inflammatory cells in the interstitium, foamy macrophages and hyalinosis in alveolar cavities, hyaline thrombi (microthrombi) in pulmonary arterioles, and atypical hyperplasia of type II alveolar epithelial cells, corresponded to typical diffuse alveolar damage. The pathological stage of transition from exudation to the organizing period was also proposed. As far as we are aware, all these detailed pathological features have not been reported in the literature so far.

It is worth noting that a de novo EGFR mutation was detected in the ILD biopsy, which was not found at the time of admission. One possible reason could be a low abundance of EGFR-mutated subclones that already existed in tumour tissue at the time of admission. These cells survived the crizotinib treatment, and their abundance increased. Recently, several studies have suggested that ROS1 rearrangements co-occur with mutations in EGFR at clinically relevant frequencies [9–11]. Another reason might be that crizotinib treatment induces acquired mutations in EGFR. Activation of EGFR, which enables cancer cells to bypass crizotinib-mediated inhibition of ROS1 signalling, has been described as a mechanism of resistance to crizotinib in ROS1-rearranged NSCLC [12]. Interestingly, in ROS1-rearranged and other fusion kinase-driven cell line models, EGFR activation and signalling appear to serve as an important early adaptive survival response to TKI exposure [13]. In this case, a decrease in original tumour lesions was observed before the time of lung biopsy, suggesting that the detectable EGFR mutations had not triggered crizotinib resistance in this period. ILD can be frequently caused by targeted therapy in EGFR-positive patients; however, the link between EGFR mutations, targeted therapy and ILD requires further investigation. It is also notable that there were no tumour cells detected in the second round of pathological diagnosis in the patient. Therefore, the detection of the EGFR mutation could be due to DNA fragments of ruptured tumour cells merging with interstices. In summary, there is not sufficient evidence to demonstrate that the EGFR mutation resulted in crizotinib resistance in this case because of radiological shrinkage of original tumour lesions and pathological features of new-onset lesion biopsy. Nevertheless, it is worth investigating the relationship between EGFR mutations and ILD in the future.

The limitations of this case report are that we did not detect hydrothorax after the treatment, and other gene mutation-detecting methods were not used to further examine all the specimens to exclude detection error.

## Conclusions

In conclusion, advanced non-small-cell lung cancer in patients with ROS1 rearrangements, treated with crizotinib, may be accompanied by fatal ILD in the initial period. Histopathology and molecular pathological examination of lung biopsy specimens is crucial for differential diagnosis and treatment guidance.

## Abbreviations

ALK: anaplastic lymphoma kinase
BRAF: B-Raf proto-oncogene
CEA: carcinoembryonic antigen
CK 7: cytokeratin 7
CT: computed tomography
EGFR: epidermal growth factor receptor
FDA: Food and Drug Administration
HE: haematoxylin-eosin
HER2: human epidermal growth factor receptor 2
ILD: interstitial lung disease
KRAS: kirsten rat sarcoma virus
MP: methylprednisolone
MPE: malignant pleural effusion
NSCLC: non-small-cell lung cancer
ROS1: c-ros oncogene 1
TKI: tyrosine kinase inhibitor
TTF-1: thyroid transcription factor-1
